# Supplementary material for: Did you donate? Talking about donations predicts compliance with solicitations for donations
Source: PLoS One. 2023 Feb 2;18(2):e0281214. doi: 10.1371/journal.pone.0281214 (PMC9894400; doi:10.1371/journal.pone.0281214)
Supplement: S1 Table — (DOCX) [file pone.0281214.s001.docx]

**S1 Table.** **Three-level structural equation model: regression of compliance on individual and collection site characteristics.**

|  | (1) | | (2) | | (3) | | (4) | |
| --- | --- | --- | --- | --- | --- | --- | --- | --- |
|  | Coef. | 95 % CI | Coef. | 95 % CI | Coef. | 95 % CI | Coef. | 95 % CI |
| **Individual level** |  |  |  |  |  |  |  |  |
| Word-of-mouth recruitment | -0.006 | [-0.028,0.015] | -0.001 | [-0.023,0.020] | -0.002 | [-0.023,0.020] | -0.001 | [-0.023,0.020] |
| Talking about donations | 0.117 | [0.095,0.140] | 0.083 | [0.061,0.106] | 0.084 | [0.061,0.106] | 0.080 | [0.049,0.110] |
| Experience | 0.007 | [0.007,0.008] | 0.007 | [0.006,0.007] | 0.007 | [0.006,0.007] | 0.007 | [0.006,0.007] |
| Talking*Experience | -0.001 | [-0.002,-0.001] | -0.001 | [-0.002,-0.001] | -0.001 | [-0.002,-0.001] | -0.001 | [-0.002,-0.001] |
| GST | -0.017 | [-0.034,-0.001] | -0.017 | [-0.038,0.002] | -0.046 | [-0.086,-0.006] | -0.018 | [-0.040,0.001] |
| Altruistic values | -0.025 | [-0.055,0.005] | -0.058 | [-0.088,-0.028] | -0.032 | [-0.048,-0.017] | -0.058 | [-0.089,-0.028] |
| Talking*Altr. values |  |  |  |  | -0.018 | [-0.049,0.012] |  |  |
| Working hours | -0.004 | [-0.005,-0.003] | -0.004 | [-0.005,-0.003] | -0.004 | [-0.005,-0.003] | -0.004 | [-0.005,-0.003] |
| Age | 0.006 | [0.004,0.007] | 0.006 | [0.005,0.007] | 0.006 | [0.005,0.007] | 0.006 | [0.005,0.007] |
| Male | 0.041 | [0.016,0.066] | 0.053 | [0.028,0.078] | 0.052 | [0.027,0.078] | 0.053 | [0.028,0.078] |
| Having children | -0.098 | [-0.125,-0.071] | -0.095 | [-0.122,-0.068] | -0.095 | [-0.121,-0.068] | -0.095 | [-0.122,-0.068] |
| Rare blood type | 0.024 | [-0.004,0.051] | 0.021 | [-0.007,0.048] | 0.021 | [-0.006,0.049] | 0.022 | [-0.006,0.049] |
| Universal blood type | 0.005 | [-0.025,0.036] | 0.002 | [-0.028,0.033] | 0.003 | [-0.028,0.033] | 0.002 | [-0.028,0.033] |
| Awareness of need |  |  | -0.003 | [-0.021,0.015] | -0.004 | [-0.022,0.015] | -0.003 | [-0.022,0.015] |
| Affective attitudes |  |  | 0.087 | [0.071,0.104] | 0.090 | [0.073,0.107] | 0.087 | [0.071,0.104] |
| Satisfaction with the BB |  |  | 0.105 | [0.078,0.133] | 0.100 | [0.074,0.127] | 0.105 | [0.079,0.132] |
| Wants more solicitations |  |  | 0.089 | [0.057,0.121] | 0.089 | [0.057,0.121] | 0.089 | [0.057,0.121] |
| Wants less solicitations |  |  | -0.432 | [-0.499,-0.364] | -0.430 | [-0.498,-0.362] | -0.432 | [-0.500,-0.365] |
| **Collection site level** |  |  |  |  |  |  |  |  |
| Prop. word-of-mouth recrt. | 0.301 | [-0.086,0.695] | 0.206 | [-0.174,0.578] | 0.199 | [-0.181,0.577] | 0.204 | [-0.179,0.583] |
| Avg. talking about donations | 0.403 | [0.052,0.750] | 0.264 | [-0.077,0.603] | 0.260 | [-0.084,0.601] | 0.269 | [-0.078,0.613] |
| Mobile | 0.019 | [-0.049; 0.087] | 0.008 | [-0.076; 0.093] | 0.006 | [-0.076,0.090] | 0.010 | [-0.072,0.095] |
| Avg. age | 0.037 | [ 0.024; 0.050] | 0.030 | [ 0.016; 0.043] | 0.030 | [0.017,0.043] | 0.030 | [0.016,0.043] |
| Prop. male | 0.826 | [0.461; 1.182] | 0.761 | [0.410; 1.108] | 0.768 | [0.416,1.112] | 0.756 | [0.405,1.105] |
| Avg. Experience | -0.011 | [-0.016; -0.005] | -0.010 | [-0.015; -0.004] | -0.010 | [-0.015,-0.004] | -0.009 | [-0.015,-0.004] |
| Prop. want more solicitations |  |  | -0.136 | [-0.548; 0.271] | -0.131 | [-0.547,0.281] | -0.145 | [-0.562,0.270] |
| Prop. Want less solicitations |  |  | -2.429 | [-3.831; -1.004] | -2.447 | [-3.853,-1.016] | -2.434 | [-3.846,-0.995] |
| Avg. Satisfaction with BB |  |  | 0.606 | [-0.288,1.676] | 0.609 | [-0.284,1.668] | 0.631 | [-0.251,1.719] |
| Talking*Mobile |  |  |  |  |  |  | 0.025 | [-0.029,0.077] |
| Constant | -2.556 | [-3.411; -1.678] | -1.811 | [-2.720,-0.891] | -1.807 | [-2.714,-0.885] | -1.807 | [-2.713,-0.890] |
| *N* | 156679 |  | 156679 |  | 156679 |  | 156679 |  |

*Notes: Coefficients are the median of the posterior distribution. 95% CI = 95% credible intervals (in brackets).*
