# Supplementary material for: Did you donate? Talking about donations predicts compliance with solicitations for donations
Source: PLoS One. 2023 Feb 2;18(2):e0281214. doi: 10.1371/journal.pone.0281214 (PMC9894400; doi:10.1371/journal.pone.0281214)
Supplement: S2 Table — (DOCX) [file pone.0281214.s002.docx]

**S2 Table.** **Three-level probit regression of compliance on individual and collection site characteristics.**

|  | (1) | | (2) | | (3) | | (4) | |
| --- | --- | --- | --- | --- | --- | --- | --- | --- |
|  | Coef. | 95 % CI | Coef. | 95 % CI | Coef. | 95 % CI | Coef. | 95 % CI |
| **Individual level** |  |  |  |  |  |  |  |  |
| Word-of-mouth recruitment | -0.007 | [-0.027,0.013] | -0.001 | [-0.022,0.019] | -0.001 | [-0.022,0.019] | -0.001 | [-0.022,0.019] |
| Talking about donations | 0.119^***^ | [0.094,0.144] | 0.087^***^ | [0.062,0.111] | 0.087^***^ | [0.063,0.112] | 0.084^***^ | [0.054,0.113] |
| Experience | 0.007^***^ | [0.006,0.008] | 0.007^***^ | [0.006,0.007] | 0.007^***^ | [0.006,0.007] | 0.007^***^ | [0.006,0.007] |
| Talking*Experience | -0.002^***^ | [-0.002,-0.001] | -0.002^***^ | [-0.002,-0.001] | -0.002^***^ | [-0.002,-0.001] | -0.002^***^ | [-0.002,-0.001] |
| GST | -0.025^**^ | [-0.040,-0.009] | -0.012 | [-0.027,0.003] | -0.012 | [-0.027,0.003] | -0.012 | [-0.027,0.003] |
| Altruistic values | -0.013 | [-0.027,0.002] | -0.030^***^ | [-0.045,-0.015] | -0.030^***^ | [-0.045,-0.015] | -0.030^***^ | [-0.045,-0.015] |
| Talking*Altr. values |  |  |  |  | -0.012 | [-0.040,0.016] |  |  |
| Working hours | -0.004^***^ | [-0.005,-0.003] | -0.004^***^ | [-0.005,-0.003] | -0.004^***^ | [-0.005,-0.003] | -0.004^***^ | [-0.005,-0.003] |
| Age | 0.006^***^ | [0.004,0.007] | 0.006^***^ | [0.005,0.007] | 0.006^***^ | [0.005,0.007] | 0.006^***^ | [0.005,0.007] |
| Male | 0.046^***^ | [0.020,0.072] | 0.055^***^ | [0.030,0.081] | 0.055^***^ | [0.030,0.081] | 0.055^***^ | [0.030,0.081] |
| Having children | -0.094^***^ | [-0.123,-0.064] | -0.092^***^ | [-0.122,-0.062] | -0.092^***^ | [-0.122,-0.062] | -0.092^***^ | [-0.122,-0.062] |
| Rare blood type | 0.026 | [-0.003,0.055] | 0.024 | [-0.004,0.052] | 0.024 | [-0.004,0.052] | 0.024 | [-0.004,0.052] |
| Universal blood type | -0.005 | [-0.036,0.026] | -0.009 | [-0.039,0.021] | -0.009 | [-0.039,0.021] | -0.009 | [-0.039,0.021] |
| Awareness of need |  |  | 0.000 | [-0.021,0.022] | 0.000 | [-0.021,0.022] | 0.000 | [-0.021,0.022] |
| Affective attitudes |  |  | 0.078^***^ | [0.061,0.096] | 0.078^***^ | [0.061,0.096] | 0.078^***^ | [0.061,0.096] |
| Satisfaction with the BB |  |  | 0.083^***^ | [0.060,0.105] | 0.083^***^ | [0.060,0.105] | 0.083^***^ | [0.060,0.105] |
| Wants more solicitations |  |  | 0.091^***^ | [0.051,0.131] | 0.091^***^ | [0.051,0.131] | 0.091^***^ | [0.051,0.131] |
| Wants less solicitations |  |  | -0.423^***^ | [-0.489,-0.356] | -0.422^***^ | [-0.489,-0.356] | -0.423^***^ | [-0.489,-0.356] |
| **Collection site level** |  |  |  |  |  |  |  |  |
| Prop. WOM recruitment | 0.322 | [-0.047,0.690] | 0.192 | [-0.167,0.558] | 0.194 | [-0.168,0.557] | 0.196 | [-0.167,0.558] |
| Avg. talking about donations | 0.408^*^ | [0.063,0.754] | 0.270 | [-0.095,0.635] | 0.271 | [-0.094,0.636] | 0.270 | [-0.095,0.635] |
| Mobile | 0.011 | [-0.066,0.089] | -0.005 | [-0.098,0.087] | -0.005 | [-0.097,0.087] | -0.005 | [-0.097,0.087] |
| Avg. age | 0.038^***^ | [0.025,0.052] | 0.031^***^ | [0.018,0.044] | 0.031^***^ | [0.018,0.044] | 0.031^***^ | [0.018,0.044] |
| Prop. male | 0.863^***^ | [0.526,1.201] | 0.829^***^ | [0.499,1.159] | 0.829^***^ | [0.499,1.159] | 0.829^***^ | [0.499,1.159] |
| Avg. Experience | -0.012^***^ | [-0.017,-0.006] | -0.010^***^ | [-0.016,-0.005] | -0.010^***^ | [-0.016,-0.005] | -0.010^***^ | [-0.016,-0.005] |
| Prop. want more solicitations |  |  | -0.153 | [-0.567,0.262] | -0.154 | [-0.568,0.261] | -0.153 | [-0.568,0.262] |
| Prop. Want less solicitations |  |  | -2.783^***^ | [-4.106,-1.459] | -2.789^***^ | [-4.111,-1.467] | -2.785^***^ | [-4.108,-1.462] |
| Avg. Satisfaction with BB |  |  | 0.248 | [-0.026,0.522] | 0.248 | [-0.026,0.522] | 0.248 | [-0.026,0.522] |
| Talking*Mobile |  |  |  |  |  |  | 0.015 | [-0.034,0.064] |
| Constant | -2.630^***^ | [-3.535,-1.725] | -2.987^***^ | [-4.272,-1.702] | -2.986^***^ | [-4.270,-1.702] | -2.986^***^ | [-4.271,-1.701] |
| *N* | 147953 |  | 145343 |  | 145343 |  | 145343 |  |

*Notes: ^*^ p < 0.05, ^**^ p < 0.01, ^***^ p < 0.001. 95% CI = 95% confidence intervals (in brackets).*
