# Supplementary material for: Did you donate? Talking about donations predicts compliance with solicitations for donations
Source: PLoS One. 2023 Feb 2;18(2):e0281214. doi: 10.1371/journal.pone.0281214 (PMC9894400; doi:10.1371/journal.pone.0281214)
Supplement: S3 Table — (DOCX) [file pone.0281214.s003.docx]

**S3 Table**. **Pearson correlation coefficients among study measures on the individual level.**

| Variables | (1) | (2) | (3) | (4) | (5) | (6) | (7) | (8) | (9) | (10) | (11) | (12) | (13) | (14) | (15) | (16) | (17) | (18) |
| --- | --- | --- | --- | --- | --- | --- | --- | --- | --- | --- | --- | --- | --- | --- | --- | --- | --- | --- |
| Compliance (1) | 1 | . | . | . | . | . | . | . | . | . | . | . | . | . | . | . | . | . |
| WOM recruitment (2) | -.04 | 1 | . | . | . | . | . | . | . | . | . | . | . | . | . | . | . | . |
| Talking about donation (3) | .08 | .02 | 1 | . | . | . | . | . | . | . | . | . | . | . | . | . | . | . |
| Working hours (4) | -.10 | .05 | -.02 | 1 | . | . | . | . | . | . | . | . | . | . | . | . | . | . |
| Having children (5) | .07 | -.10 | -.02 | -.11 | 1 | . | . | . | . | . | . | . | . | . | . | . | . | . |
| Generalized social trust (6) | -.04 | .00 | -.04 | .05 | .00 | 1 | . | . | . | . | . | . | . | . | . | . | . | . |
| Altruistic values (7) | .00 | -.01 | .10 | -.06 | -.01 | .13 | 1 | . | . | . | . | . | . | . | . | . | . | . |
| Awareness of need (8) | .08 | -.03 | .10 | -.05 | .10 | -.01 | .12 | 1 | . | . | . | . | . | . | . | . | . | . |
| Affective attitudes (9) | .15 | -.04 | .18 | -.06 | .05 | -.12 | .09 | .16 | 1 | . | . | . | . | . | . | . | . | . |
| Satisfaction with BB (10) | .10 | -.01 | .10 | -.04 | .04 | .03 | .16 | .36 | .23 | 1 | . | . | . | . | . | . | . | . |
| Wants more solicitations (11) | .03 | .00 | .05 | .03 | -.08 | -.03 | .01 | .02 | .11 | -.01 | 1 | . | . | . | . | . | . | . |
| Wants less solicitations (12) | -.09 | .01 | -.04 | .03 | .01 | .03 | -.01 | -.03 | -.08 | -.05 | -.06 | 1 | . | . | . | . | . | . |
| Age (13) | .22 | -.21 | .00 | -.27 | .46 | .00 | .02 | .16 | .12 | .07 | -.14 | .02 | 1 | . | . | . | . | . |
| Male (14) | .09 | -.03 | -.01 | .30 | .13 | .01 | -.08 | -.01 | .06 | -.06 | -.03 | .06 | .24 | 1 | . | . | . | . |
| Experience (15) | .25 | -.09 | .06 | -.06 | .22 | .02 | .00 | .13 | .14 | .02 | -.09 | .01 | .59 | .43 | 1 | . | . | . |
| Common blood type (16) | -.03 | .01 | -.02 | .02 | -.03 | .00 | .00 | -.05 | -.01 | .00 | -.02 | .01 | -.01 | .02 | .00 | 1 | . | . |
| Rare blood type (17) | .01 | -.01 | -.01 | -.01 | .01 | -.01 | .00 | -.02 | -.01 | -.01 | .03 | -.01 | .01 | -.01 | -.05 | -.71 | 1 | . |
| Universal blood type (18) | .02 | .00 | .04 | -.02 | .03 | .01 | .00 | .09 | .02 | .01 | -.01 | .00 | .01 | -.01 | .06 | -.56 | -.19 | 1 |
