# Supplementary material for: Did you donate? Talking about donations predicts compliance with solicitations for donations
Source: PLoS One. 2023 Feb 2;18(2):e0281214. doi: 10.1371/journal.pone.0281214 (PMC9894400; doi:10.1371/journal.pone.0281214)
Supplement: S4 Table — (DOCX) [file pone.0281214.s004.docx]

**S4 Table.** **Robustness checks on the predictive power of talking about donations.**

|  | (1) | | (2) | |
| --- | --- | --- | --- | --- |
|  | Coef. | 95 % CI | Coef. | 95 % CI |
| **Individual level** |  |  |  |  |
| Word-of-mouth recruitment | 0.027 | [-0.005,0.058] | 0.169^*^ | [0.026,0.313] |
| Talking about donations | 0.051^**^ | [0.015,0.087] | 0.056 | [-0.102,0.214] |
| Experience | 0.007^***^ | [0.006,0.008] |  |  |
| Talking*Experience | -0.001 | [-0.002,0.000] |  |  |
| GST | -0.012 | [-0.034,0.011] | -0.019 | [-0.095,0.056] |
| Altruistic values | -0.028^*^ | [-0.050,-0.006] | -0.158^**^ | [-0.258,-0.058] |
| Working hours | -0.004^***^ | [-0.005,-0.002] | -0.006^**^ | [-0.011,-0.002] |
| Age | 0.008^***^ | [0.006,0.009] | 0.009^*^ | [0.002,0.017] |
| Male | 0.063^***^ | [0.027,0.100] | 0.005 | [-0.190,0.201] |
| Having children | -0.070^**^ | [-0.115,-0.025] | 0.131 | [-0.055,0.318] |
| Rare blood type | 0.027 | [-0.016,0.070] | -0.014 | [-0.197,0.169] |
| Universal blood type | -0.018 | [-0.063,0.026] | -0.075 | [-0.307,0.158] |
| Awareness of need | -0.000 | [-0.029,0.028] | 0.087 | [-0.010,0.185] |
| Affective attitudes | 0.080^***^ | [0.058,0.103] | 0.223^***^ | [0.122,0.324] |
| Satisfaction with the BB | 0.048^***^ | [0.021,0.075] | 0.072 | [-0.057,0.202] |
| Wants more solicitations | 0.149^***^ | [0.092,0.206] | 0.127 | [-0.062,0.317] |
| Wants less solicitations | -0.320^***^ | [-0.409,-0.232] | -0.499 | [-1.090,0.092] |
| **Collection site level** |  |  |  |  |
| Prop. WOM recruitment | 0.398 | [-0.014,0.810] | 0.028 | [-0.317,0.374] |
| Avg. talking about donations | 0.108 | [-0.321,0.537] | 0.046 | [-0.369,0.461] |
| Mobile | -0.045 | [-0.152,0.062] | 0.089 | [-0.079,0.258] |
| Avg. age | 0.040^***^ | [0.024,0.056] | 0.005 | [-0.008,0.017] |
| Prop. male | 0.911^***^ | [0.512,1.310] | 0.181 | [-0.194,0.556] |
| Avg. Experience | -0.011^**^ | [-0.018,-0.004] |  |  |
| Prop. want more solicitations | 0.087 | [-0.420,0.595] | -0.101 | [-0.425,0.222] |
| Prop. Want less solicitations | -3.212^***^ | [-4.521,-1.903] | -0.137 | [-2.155,1.880] |
| Avg. Satisfaction with BB | 0.195 | [-0.124,0.514] | 0.046 | [-0.267,0.358] |
| Constant | -3.003^***^ | [-4.469,-1.536] | -0.547 | [-2.057,0.964] |
| *N* | 52883 |  | 3581 |  |

*Notes: The dependent variable in Model 1 is compliance with solicitations in 2013, and the sample is restricted to observations for donors that participated in the survey in 2012. In Model 2, the sample is restricted to donors that did not make any donations before participating in the survey. The robustness checks are described in more detail in section 5.2. ^*^ p < 0.05, ^**^ p < 0.01, ^***^ p < 0.001. 95% CI = 95% confidence intervals (in brackets).*
