# Supplementary material for: Did you donate? Talking about donations predicts compliance with solicitations for donations
Source: PLoS One. 2023 Feb 2;18(2):e0281214. doi: 10.1371/journal.pone.0281214 (PMC9894400; doi:10.1371/journal.pone.0281214)
Supplement: S5 Table — (DOCX) [file pone.0281214.s005.docx]

**S5 Table.** **Three-level linear probability regression of compliance on individual and collection site characteristics.**

|  | (1) | | (2) | | (3) | | (4) | |
| --- | --- | --- | --- | --- | --- | --- | --- | --- |
|  | Coef. | 95 % CI | Coef. | 95 % CI | Coef. | 95 % CI | Coef. | 95 % CI |
| **Individual level** |  |  |  |  |  |  |  |  |
| Word-of-mouth recruitment | -0.002 | [-0.008,0.005] | 0.000 | [-0.007,0.007] | 0.000 | [-0.007,0.007] | 0.000 | [-0.007,0.007] |
| Talking about donations | 0.039^***^ | [0.031,0.047] | 0.029^***^ | [0.021,0.037] | 0.029^***^ | [0.021,0.037] | 0.028^***^ | [0.018,0.037] |
| Experience | 0.002^***^ | [0.002,0.002] | 0.002^***^ | [0.002,0.002] | 0.002^***^ | [0.002,0.002] | 0.002^***^ | [0.002,0.002] |
| Talking*Experience | -0.001^***^ | [-0.001,-0.000] | -0.001^***^ | [-0.001,-0.000] | -0.001^***^ | [-0.001,-0.000] | -0.001^***^ | [-0.001,-0.000] |
| GST | -0.008^**^ | [-0.013,-0.003] | -0.004 | [-0.009,0.001] | -0.004 | [-0.009,0.001] | -0.004 | [-0.009,0.001] |
| Altruistic values | -0.004 | [-0.009,0.001] | -0.009^***^ | [-0.014,-0.005] | -0.009^***^ | [-0.014,-0.005] | -0.009^***^ | [-0.014,-0.005] |
| Talking*Altr. values |  |  |  |  | -0.003 | [-0.012,0.006] |  |  |
| Working hours | -0.001^***^ | [-0.002,-0.001] | -0.001^***^ | [-0.001,-0.001] | -0.001^***^ | [-0.001,-0.001] | -0.001^***^ | [-0.001,-0.001] |
| Age | 0.002^***^ | [0.002,0.002] | 0.002^***^ | [0.002,0.002] | 0.002^***^ | [0.002,0.002] | 0.002^***^ | [0.002,0.002] |
| Male | 0.014^**^ | [0.006,0.023] | 0.017^***^ | [0.009,0.026] | 0.017^***^ | [0.009,0.026] | 0.017^***^ | [0.009,0.026] |
| Having children | -0.030^***^ | [-0.039,-0.020] | -0.029^***^ | [-0.039,-0.020] | -0.029^***^ | [-0.039,-0.020] | -0.029^***^ | [-0.039,-0.020] |
| Rare blood type | 0.009 | [-0.001,0.018] | 0.008 | [-0.001,0.017] | 0.008 | [-0.001,0.017] | 0.008 | [-0.001,0.017] |
| Universal blood type | -0.002 | [-0.012,0.008] | -0.003 | [-0.013,0.007] | -0.003 | [-0.013,0.007] | -0.003 | [-0.013,0.007] |
| Awareness of need |  |  | 0.000 | [-0.007,0.007] | 0.000 | [-0.007,0.007] | 0.000 | [-0.007,0.007] |
| Affective attitudes |  |  | 0.025^***^ | [0.020,0.031] | 0.025^***^ | [0.020,0.031] | 0.025^***^ | [0.020,0.031] |
| Satisfaction with the BB |  |  | 0.027^***^ | [0.020,0.035] | 0.027^***^ | [0.020,0.035] | 0.027^***^ | [0.020,0.035] |
| Wants more solicitations |  |  | 0.030^***^ | [0.017,0.043] | 0.030^***^ | [0.017,0.043] | 0.030^***^ | [0.017,0.043] |
| Wants less solicitations |  |  | -0.138^***^ | [-0.159,-0.118] | -0.138^***^ | [-0.159,-0.118] | -0.139^***^ | [-0.159,-0.118] |
| **Collection site level** |  |  |  |  |  |  |  |  |
| Prop. WOM recruitment | 0.106 | [-0.014,0.226] | 0.064 | [-0.054,0.183] | 0.064 | [-0.055,0.183] | 0.064 | [-0.054,0.183] |
| Avg. talking about donations | 0.134^*^ | [0.021,0.247] | 0.086 | [-0.033,0.206] | 0.087 | [-0.033,0.206] | 0.086 | [-0.033,0.206] |
| Mobile | 0.005 | [-0.020,0.031] | 0.000 | [-0.030,0.030] | 0.000 | [-0.030,0.030] | 0.000 | [-0.030,0.030] |
| Avg. age | 0.013^***^ | [0.008,0.017] | 0.010^***^ | [0.006,0.014] | 0.010^***^ | [0.006,0.014] | 0.010^***^ | [0.006,0.014] |
| Prop. male | 0.279^***^ | [0.168,0.390] | 0.267^***^ | [0.158,0.375] | 0.267^***^ | [0.158,0.376] | 0.267^***^ | [0.158,0.375] |
| Avg. Experience | -0.004^***^ | [-0.006,-0.002] | -0.003^***^ | [-0.005,-0.002] | -0.003^***^ | [-0.005,-0.002] | -0.003^***^ | [-0.005,-0.002] |
| Prop. want more solicitations |  |  | -0.053 | [-0.188,0.082] | -0.053 | [-0.188,0.082] | -0.053 | [-0.188,0.082] |
| Prop. Want less solicitations |  |  | -0.935^***^ | [-1.377,-0.494] | -0.937^***^ | [-1.378,-0.496] | -0.936^***^ | [-1.378,-0.495] |
| Avg. Satisfaction with BB |  |  | 0.086 | [-0.005,0.177] | 0.086 | [-0.005,0.177] | 0.086 | [-0.005,0.177] |
| Talking*Mobile |  |  |  |  |  |  | 0.005 | [-0.011,0.021] |
| Constant | -0.361^*^ | [-0.652,-0.070] | -0.490^*^ | [-0.911,-0.069] | -0.490^*^ | [-0.911,-0.069] | -0.490^*^ | [-0.910,-0.069] |
| *N* | 147953 |  | 145343 |  | 145343 |  | 145343 |  |

*Notes: ^*^ p < 0.05, ^**^ p < 0.01, ^***^ p < 0.001. 95% CI = 95% confidence intervals (in brackets). Model 4 did not converge because the estimate for the random slope on talking about donations is very close to zero. It is therefore estimated using the expectation-maximization (EM) algorithm with 200 iterations.*
