# Supplementary material for: Did you donate? Talking about donations predicts compliance with solicitations for donations
Source: PLoS One. 2023 Feb 2;18(2):e0281214. doi: 10.1371/journal.pone.0281214 (PMC9894400; doi:10.1371/journal.pone.0281214)
Supplement: S6 Table — (DOCX) [file pone.0281214.s006.docx]

**S6 Table.** **OLS regression of compliance on individual and collection site characteristics.**

|  | (1) | | (2) | | (3) | | (4) | |
| --- | --- | --- | --- | --- | --- | --- | --- | --- |
|  | Coef. | 95 % CI | Coef. | 95 % CI | Coef. | 95 % CI | Coef. | 95 % CI |
| **Individual level** |  |  |  |  |  |  |  |  |
| Word-of-mouth recruitment | -0.003 | [-0.009,0.004] | -0.000 | [-0.007,0.007] | -0.000 | [-0.007,0.007] | -0.000 | [-0.007,0.007] |
| Talking about donations | 0.036^***^ | [0.028,0.044] | 0.026^***^ | [0.018,0.035] | 0.026^***^ | [0.018,0.035] | 0.025^***^ | [0.015,0.034] |
| Experience | 0.002^***^ | [0.002,0.002] | 0.002^***^ | [0.002,0.002] | 0.002^***^ | [0.002,0.002] | 0.002^***^ | [0.002,0.002] |
| Talking*Experience | -0.001^***^ | [-0.001,-0.000] | -0.001^***^ | [-0.001,-0.000] | -0.001^***^ | [-0.001,-0.000] | -0.001^***^ | [-0.001,-0.000] |
| GST | -0.007^**^ | [-0.013,-0.002] | -0.003 | [-0.008,0.002] | -0.003 | [-0.009,0.002] | -0.003 | [-0.009,0.002] |
| Altruistic values | -0.003 | [-0.008,0.002] | -0.008^**^ | [-0.013,-0.003] | -0.008^**^ | [-0.014,-0.003] | -0.008^**^ | [-0.014,-0.003] |
| Talking*Altr. values |  |  |  |  | -0.002 | [-0.013,0.009] |  |  |
| Working hours | -0.001^***^ | [-0.002,-0.001] | -0.001^***^ | [-0.002,-0.001] | -0.001^***^ | [-0.001,-0.001] | -0.001^***^ | [-0.001,-0.001] |
| Age | 0.002^***^ | [0.001,0.002] | 0.002^***^ | [0.002,0.002] | 0.002^***^ | [0.002,0.002] | 0.002^***^ | [0.002,0.002] |
| Male | 0.030^***^ | [0.021,0.039] | 0.032^***^ | [0.023,0.041] | 0.032^***^ | [0.023,0.041] | 0.032^***^ | [0.023,0.041] |
| Having children | -0.030^***^ | [-0.040,-0.021] | -0.030^***^ | [-0.040,-0.020] | -0.030^***^ | [-0.040,-0.021] | -0.030^***^ | [-0.040,-0.021] |
| Rare blood type | -0.006 | [-0.015,0.004] | -0.006 | [-0.015,0.003] | -0.006 | [-0.016,0.004] | -0.006 | [-0.016,0.004] |
| Universal blood type | -0.014^*^ | [-0.025,-0.003] | -0.015^**^ | [-0.025,-0.004] | -0.015^**^ | [-0.025,-0.004] | -0.015^**^ | [-0.026,-0.004] |
| Awareness of need |  |  | 0.000 | [-0.007,0.007] | 0.000 | [-0.007,0.007] | 0.000 | [-0.007,0.007] |
| Affective attitudes |  |  | 0.024^***^ | [0.018,0.030] | 0.024^***^ | [0.019,0.029] | 0.024^***^ | [0.019,0.029] |
| Satisfaction with the BB |  |  | 0.024^***^ | [0.017,0.031] | 0.024^***^ | [0.016,0.031] | 0.024^***^ | [0.016,0.031] |
| Wants more solicitations |  |  | 0.032^***^ | [0.018,0.046] | 0.032^***^ | [0.021,0.043] | 0.032^***^ | [0.021,0.043] |
| Wants less solicitations |  |  | -0.137^***^ | [-0.160,-0.114] | -0.137^***^ | [-0.160,-0.114] | -0.137^***^ | [-0.160,-0.114] |
| **Collection site level** |  |  |  |  |  |  |  |  |
| Prop. WOM recruitment | 0.135 | [-0.036,0.307] | 0.116 | [-0.026,0.258] | 0.116^**^ | [0.036,0.197] | 0.116^**^ | [0.036,0.197] |
| Avg. talking about donations | 0.198^*^ | [0.038,0.358] | 0.129 | [-0.023,0.281] | 0.129^***^ | [0.053,0.206] | 0.129^***^ | [0.053,0.205] |
| Mobile | 0.004 | [-0.024,0.033] | -0.015 | [-0.049,0.019] | -0.015 | [-0.030,0.000] | -0.015 | [-0.030,0.000] |
| Avg. age | 0.018^***^ | [0.013,0.024] | 0.014^***^ | [0.010,0.019] | 0.014^***^ | [0.012,0.017] | 0.014^***^ | [0.012,0.017] |
| Prop. male | 0.307^***^ | [0.164,0.451] | 0.334^***^ | [0.200,0.469] | 0.334^***^ | [0.263,0.406] | 0.334^***^ | [0.263,0.406] |
| Avg. Experience | -0.004^***^ | [-0.007,-0.002] | -0.004^***^ | [-0.007,-0.002] | -0.004^***^ | [-0.005,-0.003] | -0.004^***^ | [-0.005,-0.003] |
| Prop. want more solicitations |  |  | 0.060 | [-0.128,0.248] | 0.060 | [-0.031,0.151] | 0.060 | [-0.031,0.151] |
| Prop. Want less solicitations |  |  | -1.219^***^ | [-1.661,-0.777] | -1.219^***^ | [-1.469,-0.970] | -1.219^***^ | [-1.469,-0.970] |
| Avg. Satisfaction with BB |  |  | 0.097 | [-0.006,0.201] | 0.097^**^ | [0.035,0.160] | 0.097^**^ | [0.035,0.160] |
| Talking*Mobile |  |  |  |  |  |  | 0.008 | [-0.008,0.025] |
| Constant | -0.772^***^ | [-1.116,-0.429] | -0.849^***^ | [-1.246,-0.452] | -0.849^***^ | [-1.106,-0.593] | -0.849^***^ | [-1.106,-0.592] |
| *N* | 147953 |  | 145343 |  | 145343 |  | 145343 |  |

*Notes: ^*^ p < 0.05, ^**^ p < 0.01, ^***^ p < 0.001. 95% CI = 95% confidence intervals (in brackets). Standard errors are clustered at the collection site level.*
