# Supplementary material for: Did you donate? Talking about donations predicts compliance with solicitations for donations
Source: PLoS One. 2023 Feb 2;18(2):e0281214. doi: 10.1371/journal.pone.0281214 (PMC9894400; doi:10.1371/journal.pone.0281214)
Supplement: S7 Table — (DOCX) [file pone.0281214.s007.docx]

**S7 Table.** **Robustness checks: Alternative construction of main predictors.**

|  | (1) | | (2) | |
| --- | --- | --- | --- | --- |
|  | Coef. | 95 % CI | Coef. | 95 % CI |
| **Individual level** |  |  |  |  |
| Word-of-mouth recruitment | -0.022 | [-0.046,0.001] | -0.001 | [-0.022,0.020] |
| Talking about donations | 0.087^***^ | [0.063,0.112] | 0.094^***^ | [0.068,0.119] |
| Experience | 0.007^***^ | [0.006,0.007] | 0.007^***^ | [0.006,0.007] |
| Talking*Experience | -0.002^***^ | [-0.002,-0.001] | -0.002^***^ | [-0.002,-0.001] |
| GST | -0.012 | [-0.027,0.003] | -0.014 | [-0.029,0.001] |
| Altruistic values | -0.030^***^ | [-0.045,-0.015] | -0.029^***^ | [-0.044,-0.014] |
| Working hours | -0.004^***^ | [-0.005,-0.003] | -0.004^***^ | [-0.005,-0.003] |
| Age | 0.006^***^ | [0.005,0.007] | 0.006^***^ | [0.005,0.007] |
| Male | 0.056^***^ | [0.030,0.081] | 0.055^***^ | [0.029,0.080] |
| Having children | -0.092^***^ | [-0.122,-0.062] | -0.091^***^ | [-0.121,-0.060] |
| Rare blood type | 0.024 | [-0.004,0.052] | 0.026 | [-0.002,0.054] |
| Universal blood type | -0.009 | [-0.039,0.021] | -0.010 | [-0.040,0.021] |
| Awareness of need | 0.000 | [-0.021,0.022] | -0.000 | [-0.022,0.022] |
| Affective attitudes | 0.078^***^ | [0.061,0.095] | 0.077^***^ | [0.060,0.094] |
| Satisfaction with the BB | 0.083^***^ | [0.061,0.105] | 0.083^***^ | [0.061,0.106] |
| Wants more solicitations | 0.090^***^ | [0.050,0.130] | 0.092^***^ | [0.052,0.132] |
| Wants less solicitations | -0.423^***^ | [-0.490,-0.356] | -0.419^***^ | [-0.487,-0.352] |
| **Collection site level** |  |  |  |  |
| Prop. WOM recruitment | 0.185 | [-0.184,0.554] | 0.193 | [-0.167,0.553] |
| Avg. talking about donations | 0.284 | [-0.084,0.653] | 0.275 | [-0.093,0.643] |
| Mobile | -0.008 | [-0.099,0.083] | -0.003 | [-0.095,0.089] |
| Avg. age | 0.031^***^ | [0.018,0.043] | 0.031^***^ | [0.019,0.044] |
| Prop. male | 0.808^***^ | [0.482,1.134] | 0.827^***^ | [0.499,1.155] |
| Avg. Experience | -0.010^***^ | [-0.016,-0.005] | -0.010^***^ | [-0.016,-0.005] |
| Prop. want more solicitations | -0.163 | [-0.576,0.249] | -0.146 | [-0.562,0.270] |
| Prop. Want less solicitations | -2.825^***^ | [-4.141,-1.509] | -2.731^***^ | [-4.067,-1.394] |
| Avg. Satisfaction with BB | 0.254 | [-0.021,0.529] | 0.253 | [-0.023,0.529] |
| Constant | -2.969^***^ | [-4.243,-1.694] | -3.027^***^ | [-4.314,-1.740] |
| *N* | 145343 |  | 144573 |  |

*Notes: Model 1 uses an alternative construction of the Word-of-mouth recruitment variable indicating whether donors where only recruited via word-of-mouth, as described in section 3.2. Model 2 excludes donors that ‘often’ talk about donations, as described in section 5.2. ^*^ p < 0.05, ^**^ p < 0.01, ^***^ p < 0.001. 95% CI = 95% confidence intervals (in brackets).*
