# Supplementary material for: Did you donate? Talking about donations predicts compliance with solicitations for donations
Source: PLoS One. 2023 Feb 2;18(2):e0281214. doi: 10.1371/journal.pone.0281214 (PMC9894400; doi:10.1371/journal.pone.0281214)
Supplement: S2 Fig — AMEs are based on model 4 in S2 Table. (DOCX) [file pone.0281214.s009.docx]

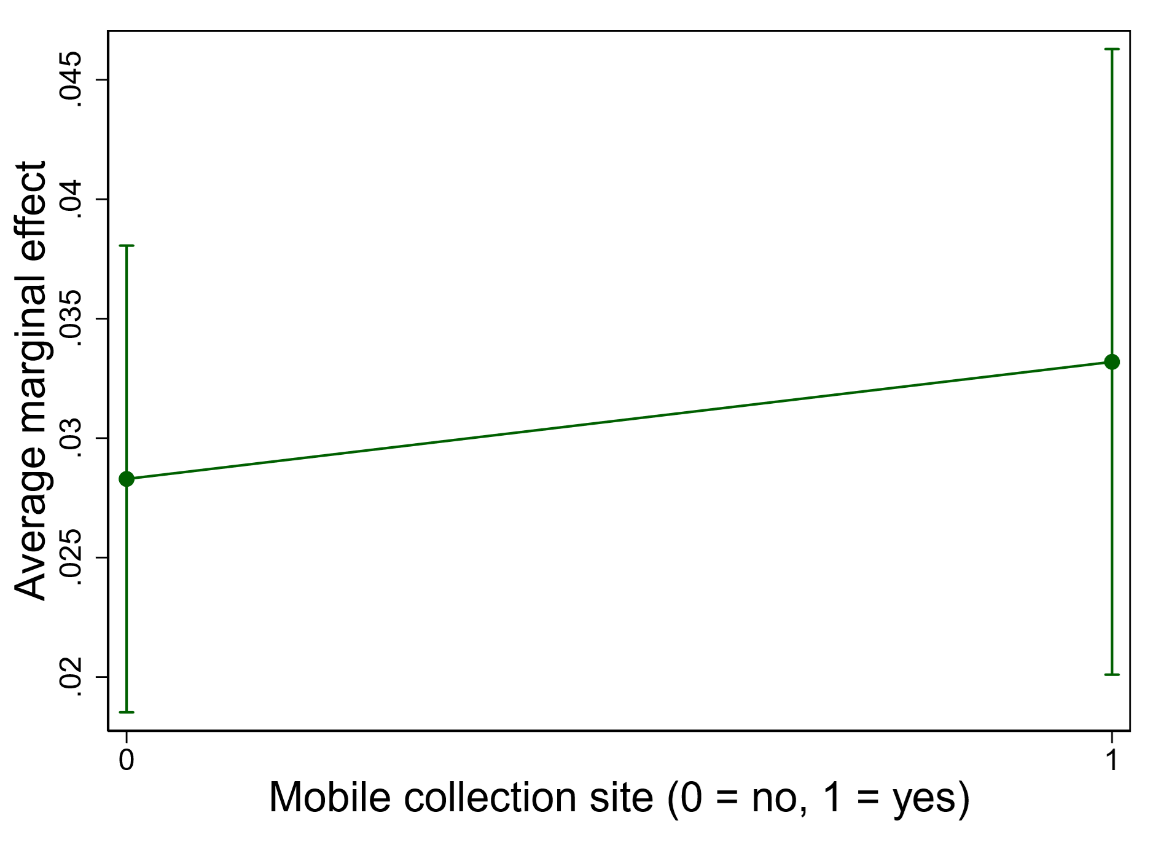


**S2 Fig. Average marginal effect of talking about donations with 95% CI by type of collection site.**

AMEs are based on model 4 in Table S2.
