# Supplementary figures and images for: Did you donate? Talking about donations predicts compliance with solicitations for donations
Source: PLoS One. 2023 Feb 2;18(2):e0281214. doi: 10.1371/journal.pone.0281214 (PMC9894400; doi:10.1371/journal.pone.0281214)

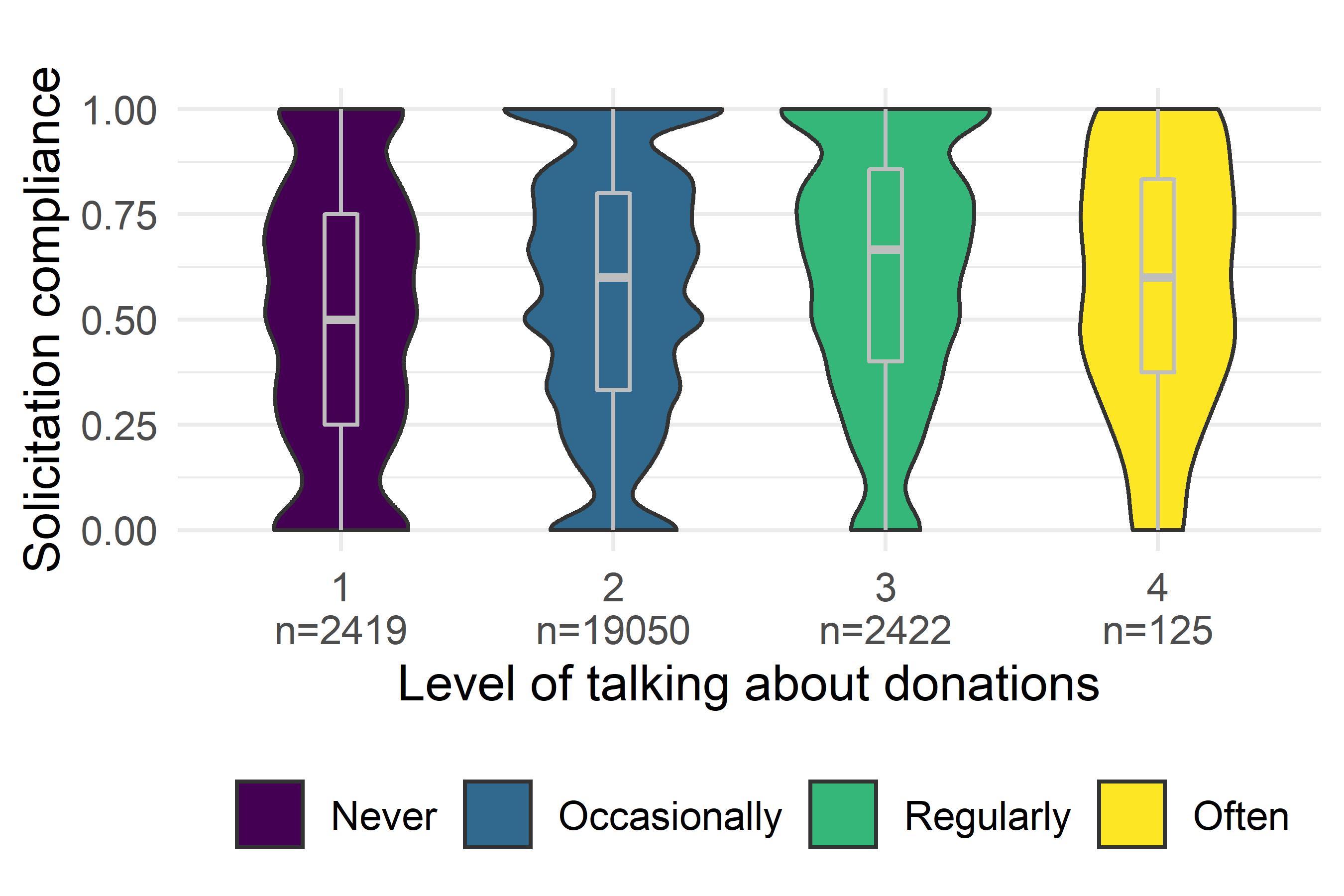

Supplement: S1 File — (TIF) [file pone.0281214.s010.tif]
