# Supplementary material for: Did you donate? Talking about donations predicts compliance with solicitations for donations
Source: PLoS One. 2023 Feb 2;18(2):e0281214. doi: 10.1371/journal.pone.0281214 (PMC9894400; doi:10.1371/journal.pone.0281214)
Supplement: Attachment — Submitted filename: Response_to_Reviewers.docx [file pone.0281214.s012.docx]

Dear Prof. Rosa-Garcia,

Thank you very much for the opportunity to revise and resubmit our manuscript entitled "Did you donate? Talking about donations predicts compliance with solicitations for donations".

Based on the comments by reviewer #1, we now refer to the paper as registered rather than preregistered.

We again would like to express our gratitude for the time and effort dedicated to improving our manuscript.

Sincerely,

Joris M. Schröder

On behalf of all authors.
